# Comparative analysis of GoPro and digital cameras in head and neck flap harvesting surgery video documentation: an innovative and efficient method for surgical education

**DOI:** 10.1186/s12909-024-05510-2

**Published:** 2024-05-14

**Authors:** Xin-Yue Huang, Zhe Shao, Nian-Nian Zhong, Yuan-Hao Wen, Tian-Fu Wu, Bing Liu, Si-Rui Ma, Lin-Lin Bu

**Affiliations:** 1 State Key Laboratory of Oral & Maxillofacial Reconstruction and Regeneration, Key Laboratory of Oral Biomedicine Ministry of Education, Hubei Key Laboratory of Stomatology, School & Hospital of Stomatology, Wuhan University, Wuhan, China; 2 Department of Oral & Maxillofacial - Head Neck Oncology, School & Hospital of Stomatology, Wuhan University, Wuhan, China

**Keywords:** Head and neck surgery, Surgery video recording, Video-based education, Medical education

## Abstract

**Background:**

An urgent need exists for innovative surgical video recording techniques in head and neck reconstructive surgeries, particularly in low- and middle-income countries where a surge in surgical procedures necessitates more skilled surgeons. This demand, significantly intensified by the COVID-19 pandemic, highlights the critical role of surgical videos in medical education. We aimed to identify a straightforward, high-quality approach to recording surgical videos at a low economic cost in the operating room, thereby contributing to enhanced patient care.

**Methods:**

The recording was comprised of six head and neck flap harvesting surgeries using GoPro or two types of digital cameras. Data were extracted from the recorded videos and their subsequent editing process. Some of the participants were subsequently interviewed.

**Results:**

Both cameras, set at 4 K resolution and 30 frames per second (fps), produced satisfactory results. The GoPro, worn on the surgeon’s head, moves in sync with the surgeon, offering a unique first-person perspective of the operation without needing an additional assistant. Though cost-effective and efficient, it lacks a zoom feature essential for close-up views. In contrast, while requiring occasional repositioning, the digital camera captures finer anatomical details due to its superior image quality and zoom capabilities.

**Conclusion:**

Merging these two systems could significantly advance the field of surgical video recording. This innovation holds promise for enhancing technical communication and bolstering video-based medical education, potentially addressing the global shortage of specialized surgeons.

**Supplementary Information:**

The online version contains supplementary material available at 10.1186/s12909-024-05510-2.

## Highlights

1) The GoPro camera offers stable vision and does not require an assistant for operation.

2) A digital camera provides images of higher quality and better anatomical detail.

3) Combining the two could result in a highly efficient and innovative method.

4) Trainees provided positive feedback on the educational impact of the videos.

**Supplementary Information:**

The online version contains supplementary material available at 10.1186/s12909-024-05510-2.

## Introduction

Innovation often occurs when knowledge from different disciplines converges and new ideas emerge or merge to foster progress [1]. Technological advancements have introduced innovations and tools that have entered head and neck surgical practice, ranging from the operating microscope and robotic, imaging-based navigation to computer-assisted design and perfusion monitoring technologies, providing precision care and better patient prognoses [1–4]. The combination of video recording and streaming with head and neck reconstructive surgery enables recording the surgeon’s view, allowing others to see exactly what the surgeon observes and does. Video recording technology can also be beneficial in various areas, such as technical communication, research, case data backup, and clinical education. As the saying goes, “A picture is worth a thousand words,” but video holds more convincing power than pictures alone. In the field of head and neck surgery, medical students and junior surgical trainees often do not acquire the full range of surgical skills during their operating room clerkships [5]. Simultaneously, the global shortage and uneven distribution of the surgical workforce are gaining recognition, with low- and middle-income countries (LMICs) in dire need of skilled surgeons [6]. There is significant demand for surgical videos in surgical education and surgeon training, especially as COVID-19 ravaged the world, affecting many residents’ clinical practice schedules to varying degrees [7, 8]. Consequently, teaching surgical skills has become more challenging.

Digital video capture during surgical procedures is an essential technology in modern-day surgical education [9–12]. The advent of fifth-generation mobile technology (5G) has facilitated the distribution of video formats, making it as effortless as sharing text and picture formats in the past, no longer constrained by mobile devices or network bandwidth. Recording surgeries in video format is employed across various domains, such as open surgery, microsurgery, laryngoscopy, and laparoscopy, yielding excellent outcomes regarding video quality and educational purposes [13–17]. Its benefits include 1) assisting medical students in their training, 2) enhancing comprehension of the surgical procedure and the patient’s clinical condition, 3) visualizing crucial routine manual operations, such as flap harvesting, 4) aiding in the preservation of legal evidence, and 5) providing a more precise anatomical description of body regions [18]. These critical aspects are challenging to convey effectively through descriptions, even with the support of photographs and other media.

The high construction costs associated with a dedicated medical recording system in the operating room can be prohibitive for some hospitals and medical institutions in LMICs and developed countries. Fortunately, due to the rapid advancement of technological innovation in recent years, personal digital video technologies have become more affordable and offer good image quality. Previous studies have also demonstrated that these technologies when applied to surgical video recording, can yield positive results [19, 20]. However, few studies have compared different types of camera systems for surgical recordings.

Our study compared the GoPro (Hero 8 Black), a low-cost commercially available action camera, with two higher-priced commercial digital cameras (Canon EOS R5 and EOS 850D). We preliminarily explored other types of surgical video recording (Figure S1), as flap harvesting is a crucial operation in head and neck reconstructive surgery with significant teaching values. Our research focused on comparing the video recording outcomes of these two camera systems during flap harvesting procedures. This study aimed to identify a straightforward, high-quality approach to recording surgical videos at a low economic cost in the operating room, thereby contributing to enhanced patient care.

## Materials and methods

The recordings were taken in the Department of Oral & Maxillofacial—Head Neck Oncology at the Hospital of Stomatology, Wuhan University, from November to December 2021. A total of six operations were prospectively recorded. All patients signed informed consent forms before surgery, and the recordings did not involve any parts of the patients’ bodies outside the operative areas.

## Devices

GoPro is a brand of action camera that can be attached to the body with simple accessories, enabling hands-free recording and first-person perspectives, especially in extreme sports. The GoPro HERO 8 Black (GoPro Inc, San Mateo, CA), used in this study, is currently a widely recognized product. This camera is exceptionally compact and portable, measuring 62*33.7*44.6 mm and weighing 450 g. The GoPro 8 supports stabilized 4 K video recording at 30 or 60 frames per second (fps) and slow-motion 1080P video at 240 fps. It is equipped with the HyperSmooth system, which stabilizes the video image without the need for external stabilizers, even when the surgeon wearing the device is moving. It can also connect to a smart device via a wireless network during filming to monitor the shot or even broadcast live using the GoPro Quik app. The fixed focus setting on this device maintains consistent focus, regardless of whether the subject gets closer or moves further away within a certain distance.

Generally, the term “digital camera” may also refer to the camera systems integrated into smartphones (such as an iPhone). However, surgical videos require precise documentation of operations on delicate anatomical structures, and our previous pilot study found that the images captured by smartphones (iPhone X) did not meet the requirements for teaching or technical communication. Therefore, the “digital camera” referenced in this article pertains specifically to professional digital cameras. We utilized two relatively recent models on the market, the EOS R5, Canon’s flagship product, and the EOS 850D, its entry-level counterpart.

## Recording

A total of six operations were prospectively studied, involving three surgeons, seven circulating nurses, and ten surgical residents.

**Fig. 1:**
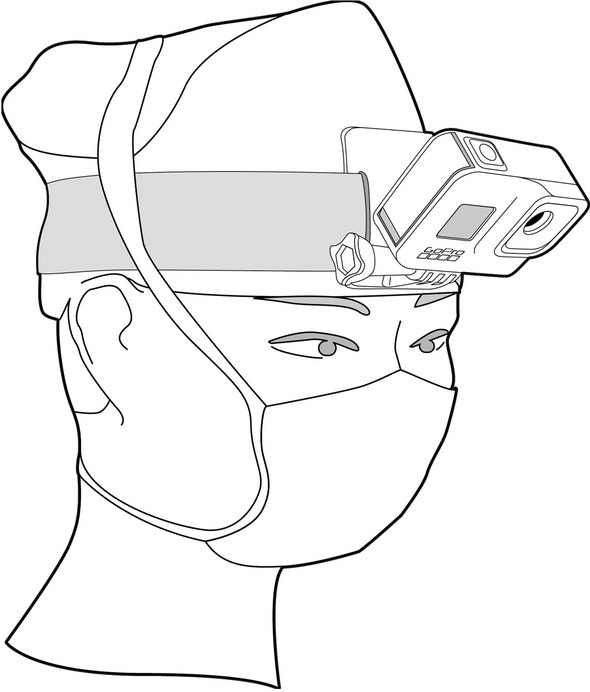
The surgeon with the head-mounted camera in place to record the surgery

The surgeon wore the GoPro 8 camera attached to a unique headband (Fig. 1), with no additional loupes or head-mounted lighting systems to physically interfere with the camera. An iPad, connected to the GoPro and equipped with the GoPro Quik app, served as a viewfinder and remote control for recording the six operations.

**Fig. 2:**
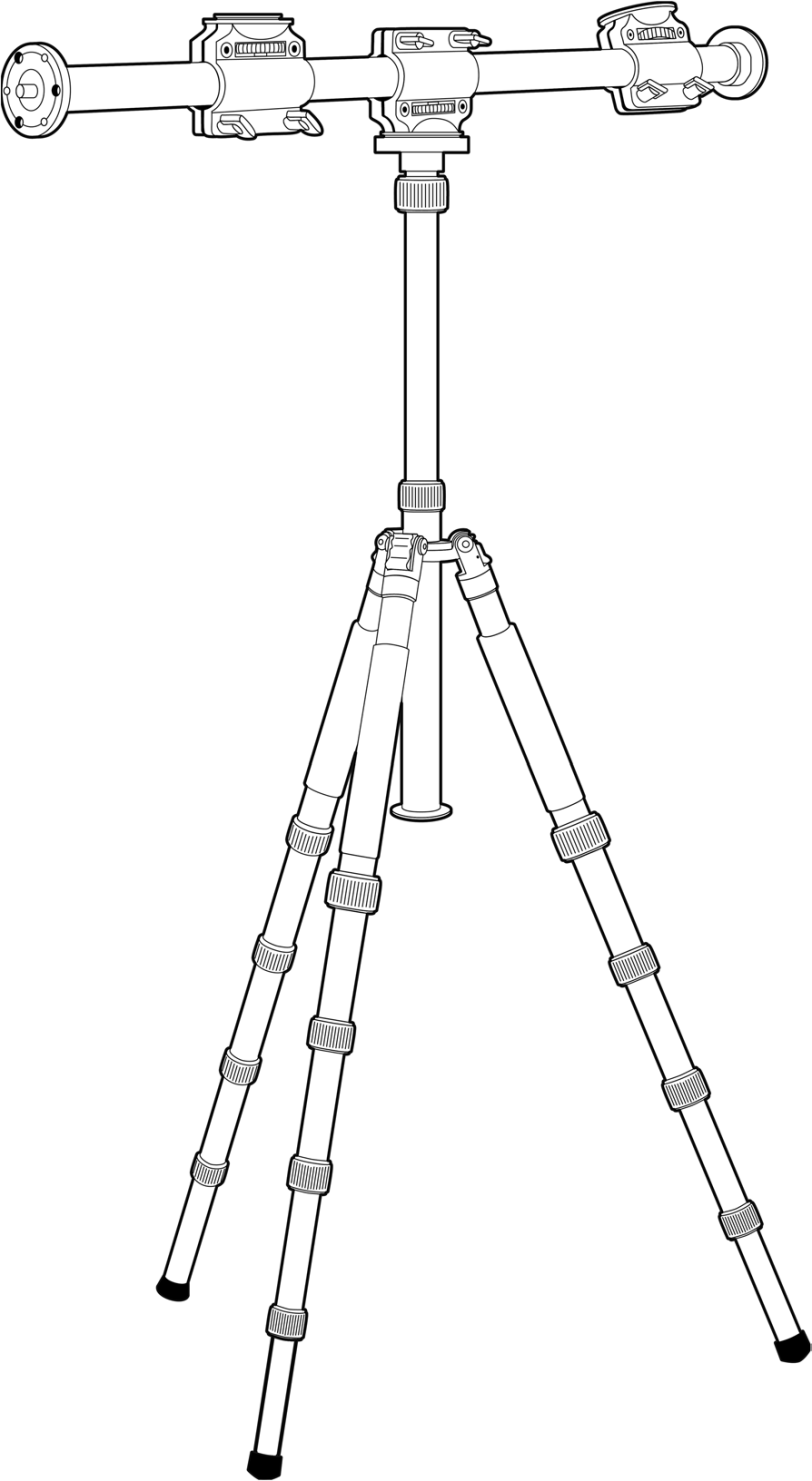
Post-assembly view of the modified tripod

The digital cameras were mounted on an external tripod for recording and were set to manual mode with manual focus. The first three recordings observed that the surgical team members occasionally obscured the surgical area. Therefore, the tripod setup was modified in the subsequent three recordings, drawing on previous studies’ methods to attain a better field of view (FOV) (Fig. 2) [21]. The sixth surgery was recorded using an EOS 850D, while the others were documented with an EOS R5.

## Characterization of videos

Data for this study were extracted from the recorded videos and their subsequent editing process. Selected variables were entered into a Microsoft Excel database. The evaluated variables included: 1) the total recording time for each device during all surgeries; 2) the duration of all surgical procedures; 3) the duration of unavailable video (including obscured surgical areas, inaccurate focus, and overexposure) and their percentage of the entire surgical procedure; and 4) the file size of the original videos.

## Educational impact analysis

To assess the quality of the recorded surgical videos and their applicability in teaching, we designed a questionnaire to gather opinions from three surgeons and nine resident physicians (students) on the edited videos. The surgeons’ questionnaire primarily assessed whether the videos clearly conveyed the surgeon’s operational concepts and specific details. Trainees were queried whether they could clearly view the procedures and derive educational benefits from it.

To further analyze the educational impact of the surgical videos, we established an Expert Review Panel (ERP) comprising five experts with over ten years of experience in clinical surgery and medical student education. We also created an assessment table for evaluating surgical education video (Table 1). The ERP reviewed six edited surgical videos and evaluated their instructional quality, clarity, stability, and effectiveness in conveying surgical techniques. Subsequently, the table was completed to categorize the overall quality of each video.

**Table 1:** Expert review panel assessment criteria

	**QUALITY CATEGORY 1 ** **High-quality surgical videos available for clinical teaching application** **Video described by all ** **of the below**	**QUALITY CATEGORY 2** **Surgical videos available for clinical teaching application** **Video described by any ** **of the below**	**QUALITY CATEGORY 3** **Surgical videos available for clinical teaching application, but need a better replacement** **Video described by any ** **of the below**	**QUALTIY CATEGORY4** **Objection to clinical teaching application** **Video described by any ** **of the below**
Video clarity	Fully acceptable	Acceptable but missing little details, not affect watching	Acceptable but missing details, likely to have an impact on watching	Unacceptable with lots of details missing
Video stability	Fully acceptable	Acceptable but with very small amount of shaking, not affect watching	Acceptable but with part of shaking, likely to have an impact on watching	Unacceptable with a large amount of shaking
Effectiveness in conveying surgical techniques and procedures	Completely convey the technical details and operation procedures of the surgeon	Very likely to convey the technical details and operation procedures of the surgeon	Likely to convey the technical details and operation procedures of the surgeon	Very unlikely to convey the technical details and operation procedures of the surgeon
Instructional quality	Very likely to benefit the medical students who watch the video for learning surgical techniques	Likely to benefit the medical students who watch the video for learning surgical techniques	Unlikely to benefit the medical students who watch the video for learning surgical techniques	May mislead the medical students who watch the video for learning surgical techniques

## Results

It took approximately 10 min to prepare the video equipment before surgery, including setting up the tripod, determining the recording settings, and adjusting the camera’s position. All surgeons reported that the head-mounted recording device did not interfere with the operation. The operations involved different types of flap harvesting, including ilium flap harvesting (*n* = 3), fibula flap harvesting (*n* = 1), anterolateral thigh flap harvesting (*n* = 1), and forearm flap harvesting (*n* = 1). The average duration of the operations was one and a half hours. Six surgical procedures were recorded simultaneously using both the GoPro and digital cameras. The characteristics of each surgical video are shown in Table 2. The technical details of the two types of cameras used in the study (GoPro HERO 8 Black, EOS R5, and EOS 850D) are summarized in Table 3. A video of the sample can be found in the Supplementary Video.

**Table 2:** Characteristic statistics of six surgical videos

Operation	Devices	Position	Total file size (video time)	Time of surgical procedure	Duration of unavailable video (percentage)
Ilium flap harvesting 1	EOS R5	Tripod	48.1 GB (119′22’’)	113′32’’	51′46’’ (45.6%)
Forearm flap harvesting	EOS 850D	Handheld	54.5 GB (65′41’’)	65′25’’	12′49’’ (19.6%)
Anterolateral thigh flap harvesting	GoPro	Surgeon’s head	22.5 GB (58′55’’)	55′41’’	1′03’’ (1.9%)
	EOS R5	Tripod	26.8 GB (66′31’’)	63′59’’	18′53’’ (29.5%)
Ilium flap harvesting 2	GoPro	Surgeon’s head	42 GB (99′59’’)	99′16’’	2′27’’ (2.5%)
	EOS R5	Modified tripod	40.5 GB (100′25’’)	99′15’’	14′38’’ (14.7%)
Ilium flap harvesting 3	GoPro	Assistant’s head	21.6 GB (51′28’’)	51′00’’	13′49’’ (27.9%)
	EOS R5	Modified tripod	45.7 GB (113′30’’)	113′16’’	15′36’’ (13.8%)
Fibula flap harvesting	GoPro	Surgeon’s head	47.2 GB (112′18’’)	111′01’’	2′10’’ (2.0%)
	EOS R5	Modified tripod	49.4 GB (122′25’’)	122′20’’	6′44’’ (5.5%)

**Table 3:** Specifications of three cameras

	GoPro Hero 8	EOS R5	EOS 850D
Video format	MP4 (H.264)	MP4/RAW (H.265/H.264)	MP4 (H.264)
Sensor	1/2.3" CMOS	36*24 mm CMOS	22.3*14.9 mm CMOS
Video resolution (max)	12 million active pixels	45 million active pixels	24.1 million active pixels
Recording Format	4 K Ultra HD video: 3840*2160 24/25/30/48/50/60p	8 K UHD: 8192* 5464 24/25/30p	4 K UHD: 3840*2160 24/25/30/48/50/60/120p
	2.7 K HD: 2704*1520 24/25/30/48/50/60p	4 K UHD: 3840*2160 24/25/30/48/50/60/120p,	Full HD: 1920*1080 24/25/30/48/50/60/120p
	Full HD: 1920*1080 24/25/30/48/50/60/120p	Full HD: 1920*1080 24/25/30/48/50/60/120p	
Connectivity/HD monitor connection	Wi-Fi + Bluetooth	802.11 a/b/g/n/ac, 2.4/5 GHZ dual-band Wi-Fi + Bluetooth 5.0; HDMI cable	Wi-Fi + Bluetooth; HDMI cable
Storage	MicroSD slot up to 128 GB	SD card slot up to 128 GB	SD card slot up to 128 GB
Lens/Field of view	Ultrawide/Wide/Linear/Narrow	RF 24-105 mm f/4L IS USM	EF 24-105 mm f/4L IS II USM
Weight (g)	450 g	650 g (body only), 738 g (including battery and memory card)	471 g (body only), 515 g (including battery and memory card)
Dimensions w *h*l	62*33.7*44.6 mm	138.5*97.5*88 mm	131*102.6*76.2 mm
Battery (mAh)	1220mAh	LP-E6N(1865mAh)/LP-E6(1800mAh)	LP-E17(1040mAh)
Support	Commercial, not for medical use head belt	Modified tripod	Modified tripod
Voice control	O	X	X
Water Proof	O	X	X
Reprocessing	Soakable, Povidone-iodine sterilization	Non-medical device	Non-medical device
Price/Cost	$399.00	$3,899	$749.99

## Video settings

**Fig. 3:**
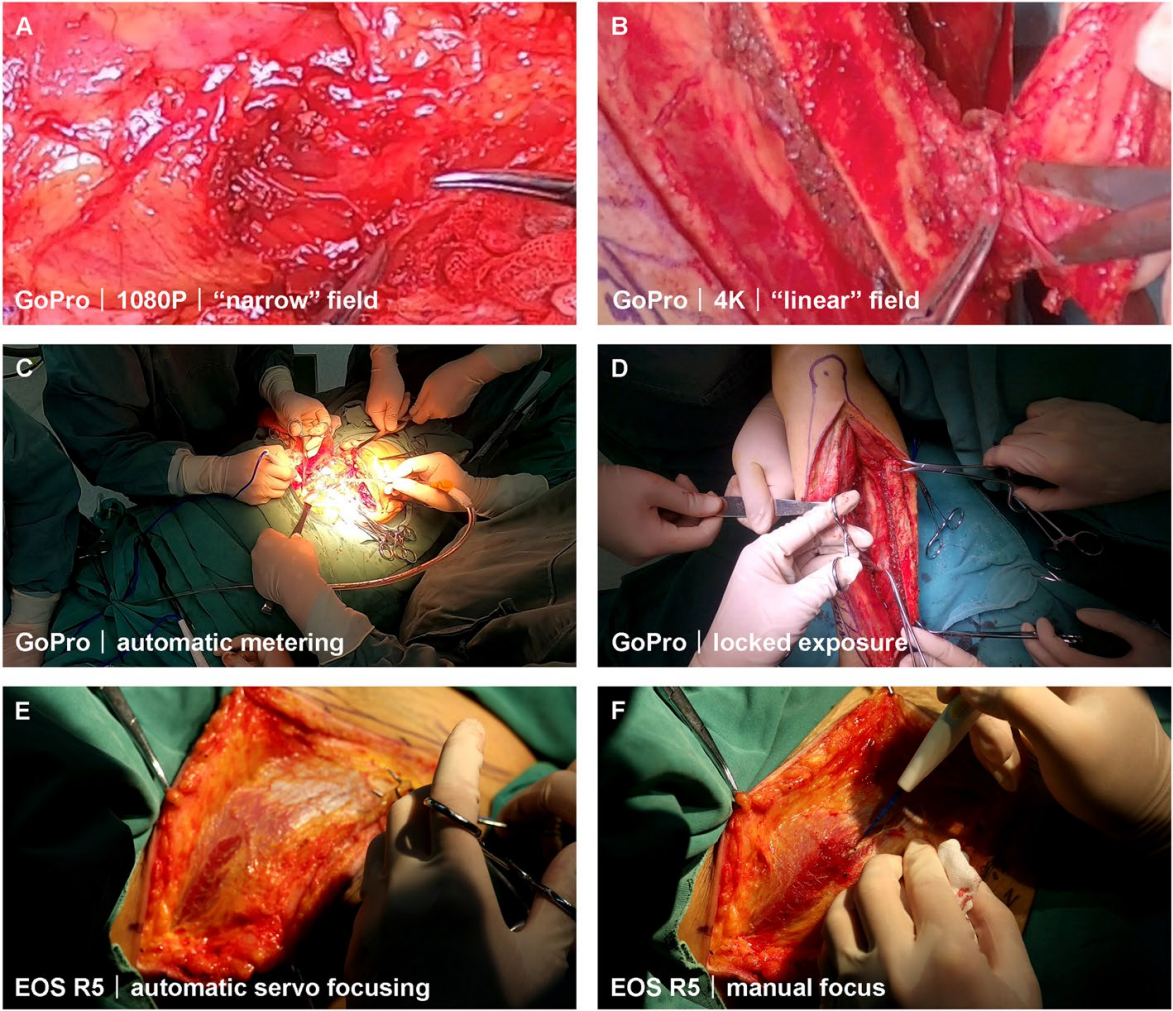
Image quality comparison of video screenshots with the magnification of 500% obtained from (**A**) 1080P narrow field and (**B**) 4K linear field. **C** When set to automatic metering, the intraoperative area was overexposed, and (**D**) the area was normally exposed after locked exposure. **E** In automatic servo focusing mode, the focal plane is not in the area but in the operator’s hand. **F** Manual focus keeps the focus on the area, even if the operator’s hand is covered

While filming surgeries, we consulted previous studies for camera settings. Graves et al. conducted research using a GoPro camera in the operating room [20]. They selected an earlier type—the GoPro HERO 3 + Black—and concluded that with a narrow FOV, automatic white balance, 1080P resolution, and 48 fps, one could achieve high-quality, low-cost video recordings of surgical procedures. In our study, we initially tried a 1080P resolution with a narrow field and obtained relatively good results (Fig. 3A). However, a 1080P resolution HD video consists of two million pixels (1920 × 1080), whereas a 4 K (Ultra HD) video comprises over eight million pixels (3840 × 2160). Thus, 4 K video produces a sharper image with four times the resolution of 1080P. Given that the GoPro does not support narrow-field shooting at 4 K resolution, we discovered that setting it to a “linear” FOV with 4 K resolution provided more precise and crisper imagery (Fig. 3B). In most of our recordings, we used 4 K resolution and a frame rate of 30 fps for both the GoPro and digital cameras.

Light metering involves determining the necessary exposure based on environmental conditions, which can be manually adjusted by the photographer with different exposure settings or automatically by the camera’s program. The light environment of the operating room is complex, as the surgical field illuminated by a shadowless lamp is typically brighter than the surrounding area. When recording the surgical area with a digital camera that permits manual operation, it is recommended to use a smaller aperture (an opening that allows light to reach a lens) to reduce light intake and increase the depth of field (DOF), which is the range within which objects appear sharp. Although the GoPro was set to automatic metering mode due to the difficulty of manual operation, its FOV shifted with the surgeon’s head movements. As bright lights continually focused on the surgical area, rapid changes in the FOV easily caused overexposure in the operating area (Fig. 3C). This issue was later addressed by locking the exposure before recording (Fig. 3D).

The first recorded video revealed that the digital camera’s automatic servo focusing caused instability in the focal plane within the operational area due to various instruments and the surgeon’s hands in the surgical field (Fig. 3E). This issue was addressed by manually focusing and locking the focal plane before recording (Fig. 3F). However, when the position changed during the procedure, an assistant without surgical hand disinfection was required to adjust the camera promptly.

## Quality of videos

The image quality of videos from three cameras was sufficient for depicting static and moving objects. However, the operating room is a unique environment where multiple factors influence the cameras’ effectiveness. These factors include the distance from the operating area, obstruction by surgical team members [22], the lens’s FOV, light overexposure, and reflection from metal instruments.

**Fig. 4:**
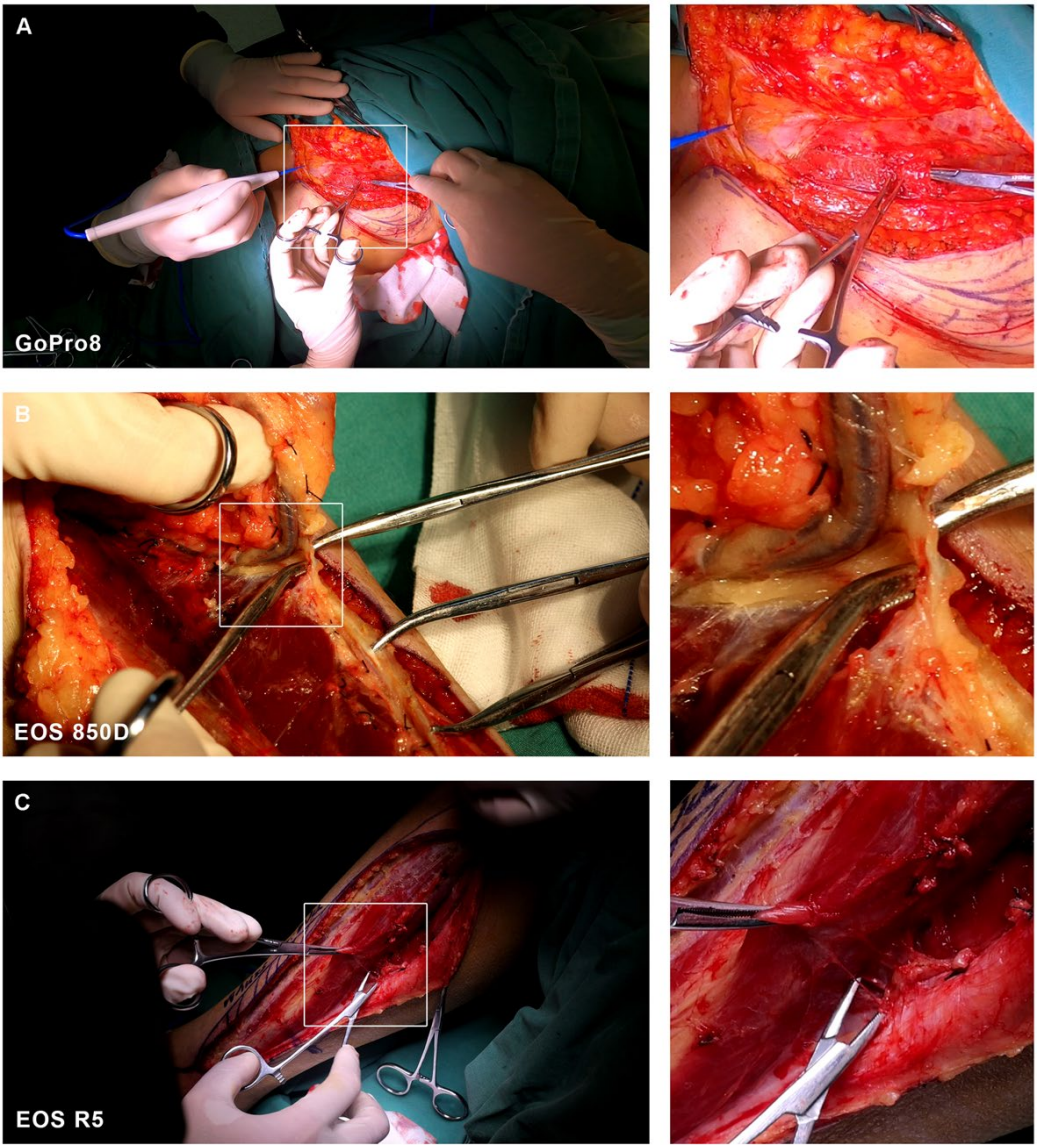
**A** 100% image from uncompressed file video of GoPro8, compared with magnified 300% video image in detail. **B** 100% image from uncompressed file video of EOS 850D, compared with magnified 300% video image in detail. **C** 100% image from uncompressed file video of EOS R5, compared with magnified 300% video image in detail

To more clearly compare the video quality of the three devices across six different head and neck reconstructive procedures, we extracted images from the video files of all devices and assessed their clarity at magnifications of 100% and 300%. Fig. 4A-C show 100% images alongside detailed 300% magnified images captured from videos recorded by the GoPro8, EOS 850D, and EOS R5, respectively. All three devices provided precise and reliable output under various circumstances and lighting conditions.

## Positioning in the operative room

Digital cameras capture high-definition images during surgery with accurate focus. However, there were instances when the lens was obscured by the bodies of surgical team members or instruments, missing critical moments. As shown in Table 2, videos recorded with a digital camera placed on an unmodified tripod had a higher rate of unavailable video duration, primarily due to obscuration. The tripod was modified to position the camera’s FOV more perpendicular to the surgical area. Consequently, the average duration of unusable video recorded by the digital camera using the modified tripod was 11.3%, a significant decrease from the 37.55% average without modification.

**Fig. 5:**
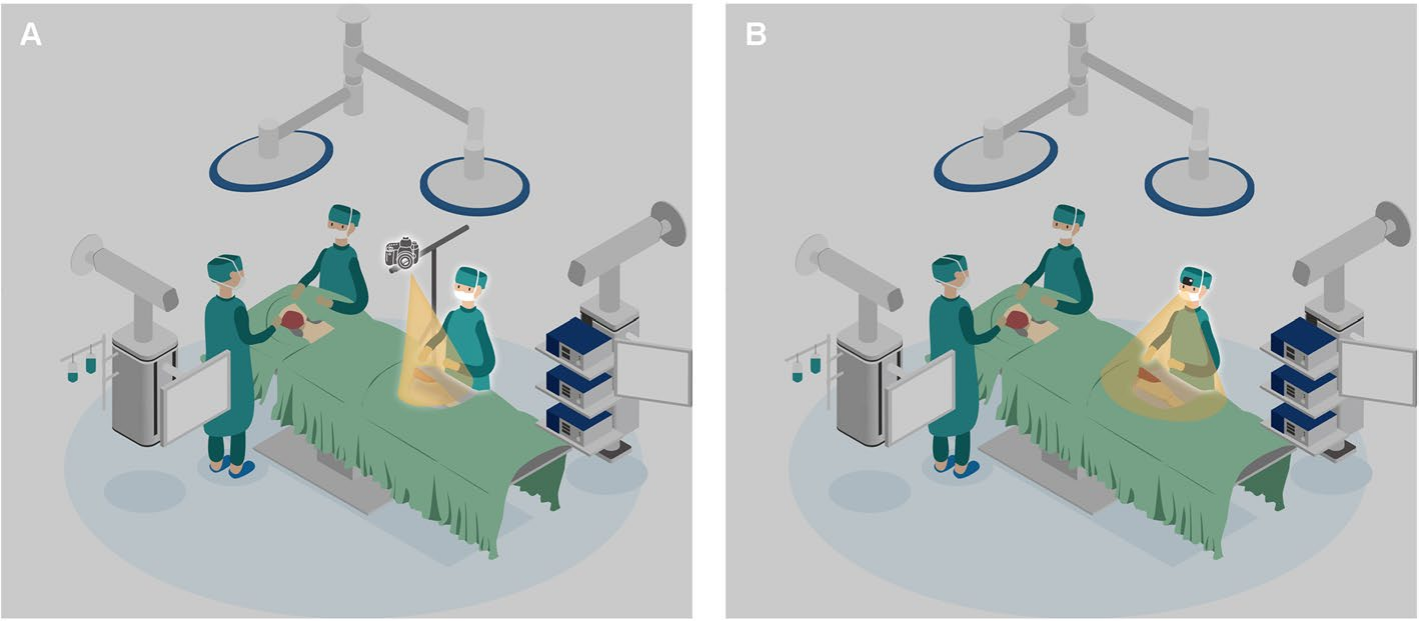
The position used for the recording with the surgical field and surgeon. **A** Ilium flap harvesting with digital camera. **B** Ilium flap harvesting with GoPro

Figure 5 depicts the positioning of the modified tripod, head-mounted camera, and surgical field used for the recording.

## Field of view

In the recordings of Anterolateral thigh flap harvesting, Ilium flap harvesting 2, and Fibula flap harvesting surgeries, the use of the GoPro resulted in a lower duration of unavailable video (1.9%, 2.5%, and 2.0%, respectively) compared to digital cameras (29.5%, 14.7%, and 5.5%), even with the tripod modified for the latter two recordings. This outcome is primarily because the surgeon’s hand often blocked the digital camera, positioned for a third-person perspective. In contrast, the GoPro, attached to the surgeon’s head, offered a viewpoint closer to the surgeon’s own eyes, thereby capturing a better visual field. The surgeon’s perspective is arguably the most advantageous, as corroborated by many previous studies that placed the camera on the surgeon’s forehead for procedural recording [13, 20, 23]. Rafael et al. reported that the head camera position was well-received by volunteers [24]. Though we obtained valuable images this way, there were limitations. The angle from the eye to the target point varies with the surgical techniques. Digital cameras can easily shift focus by adjusting the tripod’s position and angle to maintain the view of the surgical area; however, the GoPro’s FOV and focus are fixed upon installation, allowing horizontal adjustments, occasionally resulting in the surgical area going out of frame. Nevertheless, the GoPro’s wide FOV in both “wide” and “linear” modes generally ensures that the area remains within the shot without continuous monitoring.

Based on the images extracted from the videos, the digital camera can achieve a more detailed view of the surgical area with its zoom capabilities compared to the GoPro’s wider FOV. Although the GoPro’s images reveal clear anatomical structures upon magnification, they are not as sharp as those from the digital camera. This limitation, however, had unexpected benefits, as it could record the surgeon’s hand movements between the patient’s tissues and the instruments, providing insights into surgical hand positioning and instrument ergonomics that are crucial for training but often overlooked [23]. Experienced surgeons efficiently organize their workspace, holding instruments currently in use while preparing others for subsequent steps. On-site trainees, focusing primarily on the operative site, may miss these subtle ergonomic maneuvers. When used in education, surgical recordings simultaneously displaying the operative site and hand positioning can offer learners vital insights previously unnoticed [25].

## Connectivity

**Fig. 6:**
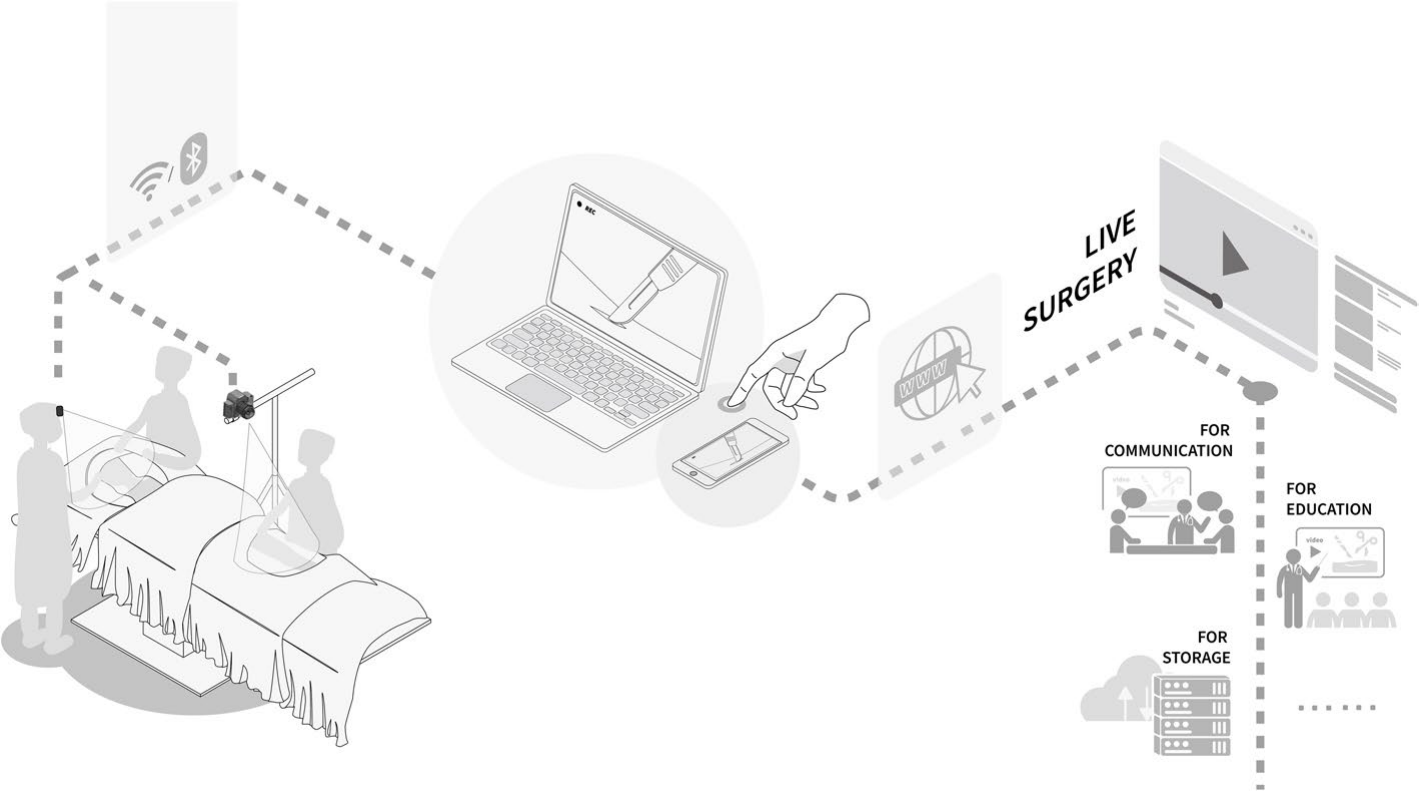
The camera can be connected to a mobile phone or laptop via Wi-Fi or Bluetooth or even broadcast live via the Internet for more purposes

All three devices possess the capability for wireless connectivity via Wi-Fi or Bluetooth systems. Video captured by these devices can be streamed in real-time to nearby mobile devices or monitors and can even be broadcast online. This feature forms the foundation for remote tele-proctoring and education purposes in surgery, a method proven to be innovative for enhancing surgical education in high-resource settings [26]. Fig. 6 illustrates the connectivity scheme, which includes a wireless link between the cameras and mobile devices through Wi-Fi or Bluetooth, facilitating further dissemination by these devices.

In addition, the GoPro device itself comes equipped with a Livestream function in full 1080P HD mode. However, the video quality of the Webcast is not as high as the recordings due to limitations imposed by wireless connection speeds and bandwidth. The "choppy" nature of the video presentation during streaming can be mitigated by using a direct cable for live broadcasts, allowing direct streaming onto a monitor for local presentation or broader live broadcasts, and offering a quality superior to Wi-Fi or Bluetooth options. The downside is the cumbersome nature of the required cables.

## Editing of video

Benefiting from the high resolution of 4K video, high definition is maintained even after the original video clip is magnified. Structural details are well-preserved, and the clarity of the operation remains evident in the magnified version, which can be further saved or shared. GoPro Quik, an application developed by the GoPro company, facilitates customized video editing. It can be used to edit original clips shot by the GoPro camera, re-adjust the field of interest, and conveniently export the video in the appropriate format. High-resolution video has its pros and cons. The extensive data involved makes storing and editing raw video files challenging. Future technologies should enable surgeons to ensure real-time recording of the area of interest, allowing for more manageable data acquisition without the need for zooming or cropping post-capture.

## Videos for education

**Fig. 7:**
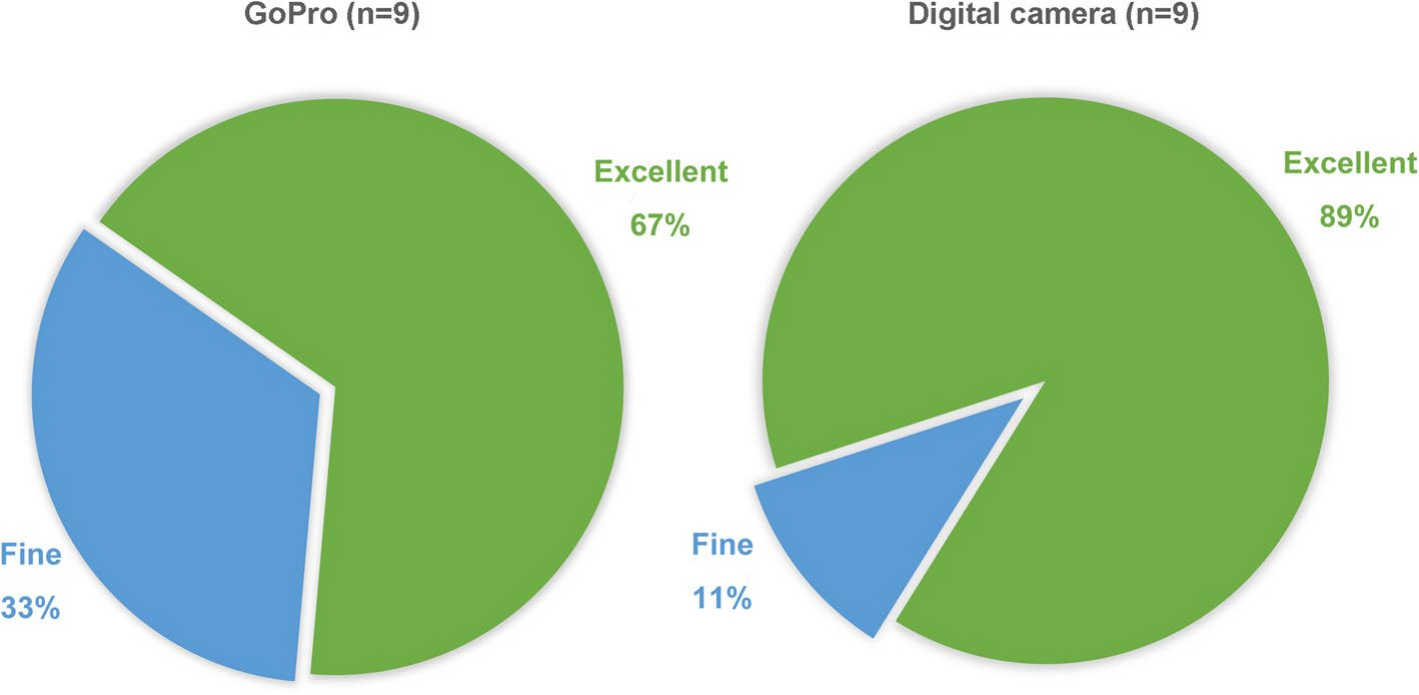
Trainees reported satisfaction degrees from surgical videos, according to whether they can see the procedure clearly and learn from it—comparison of GoPro versus digital camera

The results of the questionnaire were as follows. In the surgeons’ group, 100% (*n* = 3) confirmed that the videos well represented the details of their operations. In the students’ group, 66.7% of respondents (*n* = 6) rated the image quality with GoPro as excellent, and 33.3% (*n* = 3) found it fine, while for the digital camera, 88.9% of respondents (*n* = 8) rated it as excellent and 11.1% (*n* = 1) as fine (Fig. 7). All respondents (*n* = 9) positively affirmed that they could learn professional skills from the videos. In the evaluation conducted by the Expert Review Panel, of the six videos, four were considered suitable for clinical teaching applications, one was also suitable but required a better replacement, and one was deemed unsuitable for clinical teaching applications. However, these results must be interpreted with caution due to the small sample size.

## Discussion

With the increasing demand for technical communication, medical teaching, surgical procedure recording, and so on, surgical video has become a popular multimedia mode. It is a powerful medium that can enhance patient safety in several ways: education, real-time consultation, research, process improvement, and workflow coordination [27]. Operation videos can be transmitted through the internet in real-time, providing a platform for communication and cooperation between hospitals. Experienced surgeons can assess trainees’ surgical competency in an unbiased fashion through the trainees’ intraoperative video [28]. Experienced individual surgeons hope to share their professional knowledge and skills through surgical videos and achieve the purpose of self-publicity. Regarding privacy protection, Turnbull et al. emphasized that video documentation has significant ethical and legal considerations as it contains personal information and infringes on patients’ privacy [29]. The patient’s privacy should be carefully considered to avoid potential ethical and legal conflicts brought about by filming operations.

## Pros and cons of two camera systems

The introduction of video technology into surgical procedures is becoming more common, and high-resolution camera technology has been integrated into surgical instrumentation for laparoscopic and minimally invasive procedures [30]. Although technology continuously evolves, leading to the adoption of many new technologies in intraoperative video recording, there are still limitations in devices for capturing open surgery. Due to economic conditions and space constraints, operating rooms are not routinely equipped with video recording equipment, making personal recording equipment a more viable solution. This study compared two technologies (GoPro and digital camera) used for intraoperative video capture in open surgeries and summarized their advantages and disadvantages (Table 4).

**Table 4:** Pros and cons between GoPro and digital cameras

	GoPro	Digital camera
Advantages	HD picture quality Portable Stabilization Remote control live streaming waterproof Low price Better FOV	HD picture quality More accurate vision Remote control More maneuverability Relatively low prices
Disadvantages	No zoom Lack of maneuverability Small battery capacity The fuselage fever	A tripod is required High requirements for recording position Need assistant operation

GoPro cameras are designed for extreme sports, featuring high resolution, high frame rates, small image sensors, and a lack of complete manual control. They are light and portable enough to be worn on a surgeon’s head, providing an image that approximates the natural field of vision without hindering the operation. Simultaneously, their built-in stabilizer function ensures the output image remains stable and visible. Being waterproof, they can be soaked in povidone-iodine for disinfection, facilitating hand-held shooting [31]. Existing studies confirm that this disinfection method does not compromise asepsis [32]. The built-in Wi-Fi and Bluetooth allow for remote monitoring and real-time transmission of intraoperative video. The affordability of GoPro enables doctors wanting to record surgeries to do so cost-effectively, making them accessible to surgeons from LMICs. However, the downsides are clear: users in the operating room are more likely to obtain a narrower FOV aimed at the surgical area, but the GoPro, as an action camera, is designed to capture as comprehensive a panoramic view as possible. Due to the absence of manual controls, it does not adapt well to frequent brightness changes caused by bright overhead operating room lights. Additionally, the battery capacity of the GoPro lasts approximately 60 min and may shut down if its body temperature reaches an upper limit during prolonged sessions. For extened recording times, spare batteries are necessary, and consideration of the device temperature is essential.

Digital cameras, due to their optimal optical performance and excellent zoom capabilities, can capture specific areas of interest in high quality. They are typically more durable and are generally equipped with larger image sensors, better adapting to unfavorable lighting conditions. Their robust maneuverability makes them suitable for the complex operating room environment. Digital cameras were not widely used for surgical video recording due to their high cost. However, this study shows that even inexpensive digital cameras, such as the EOS 850D, can produce adequate surgical videos. The picture quality is not significantly different from the much pricier EOS R5 when using the same 4 K 30 fps model. Of course, more expensive cameras like the EOS R5, which supports 8 K quality video, allow for a better representation of delicate anatomy.

Nevertheless, these cameras’ drawback is that they always require supports like tripods or rocker arms for steady recording. The positioning, height, and relationship with the surgical team determine the final video quality. Furthermore, an additional assistant is needed to adjust camera positions and video settings to maintain the appropriate shooting angle during the procedure. This camera operator might need to direct the surgeon to stop and start at different points throughout the surgery, potentially interfering with the surgical team. The risk of breaking sterility should also be considered when introducing an extra individual into the operating room. This cumbersome and time-consuming shooting method does not lend itself to daily, routine intraoperative videotaping.

Using either a GoPro or a digital camera is a commendable choice. According to our research, the GoPro is a highly efficient option that is better suited for personal recording and can be operated easily without an assistant. Digital cameras, though requiring additional assistance, deliver higher output quality. If the two are innovatively combined, images from different fields of vision can be captured to produce rich, comprehensive, and high-quality videos.

## Application of surgical video in education and other aspects

Surgical video holds broad application prospects in medical teaching, technical communication, patient safety, workflow coordination, case data backup, research, real-time consulting, and skill improvement. With the advancement of communication facilities, real-time video recording during surgery presents extensive development prospects akin to digital twin technology [33, 34]. Mentoring through this medium can enhance quality and patient safety throughout a medical student’s career. Future developments may involve coaching sessions or honing non-technical skills, such as optimizing teamwork in the operating room to elevate patient care.

Medical students’ journey to becoming surgeons critically requires specific technical feedback while developing foundational skills during their internships. Despite the importance of targeted feedback, medical students often endure inconsistent, fragmented, and stressful experiences in the operating room [5]. Compounding these challenges, a study on oral and maxillofacial surgery trainees in the United States revealed that the COVID-19 pandemic disrupted the scheduling of non-urgent and elective operations [35]. With approximately 2.28 million more skilled medical professionals needed to meet the global demand for surgical procedures [6], training a substantial cohort of future surgeons is a pressing, worldwide challenge.

In addition to traditional book learning and clinical practice, watching surgical videos can help medical students acquire technical details related to surgical operations more precisely, and some critical but fleeting points can be repeated during video playback. Video-based interventions to enhance surgical skills are gaining attention for their educational applications and related research [12]. The use of video technology in teaching is relatively common in other fields, including sports. In head and neck surgery, some advantages of utilizing high-quality surgical recordings as educational tools are as follows: 1) They provide clear, sharp images that depict fine anatomical structure; 2) Learning through videos offers a more intuitive experience, as viewing surgery footage from a first-person perspective affords residents a more immersive sensation, encouraging them to conceptualize the surgery from the surgeon’s viewpoint; 3) Video recordings of resident physicians’ operations facilitate the assessment of their skill levels, paving the way for enhanced performance; 4) Essential intraoperative findings can be documented and elucidated; 5) The zoom feature enables close-up, detailed recording of surgical procedures and anatomical nuances.

Leveraging the Wi-Fi and Bluetooth capabilities of recording devices, real-time videos can be streamed to mobile phones or laptops or even broadcast live over the internet for tele-proctoring. This emerging technology allows instructors to provide real-time guidance and technical support through audio and video interactions from various geographical locations. This method effectively circumvents the additional logistical costs, time constraints, and challenges posed by distance that are inherent when instructors physically travel to the field [36]. McCullough et al. [26] previously explored the feasibility of wearable recording technology in expanding the reach and availability of specialized surgical training in LMICs, using Mozambique as a case study. Their research suggests that this educational model connects surgeons globally and fosters advanced mentoring in regions where surgical trainees have limited opportunities.

## Limitations

The findings of this study must be considered within the context of certain limitations. The research was single-centered with a limited number of surgeons involved, and only a single brand of digital camera was selected, which may lead to a lack of diversity and overlook ergonomic differences between types of surgeries and the subtle imaging details between different camera manufacturers. The assessment of the impact of video on teaching also had a small sample size, so potential biases in questionnaire feedback should be considered. Furthermore, there is a persistent need for objective and repeatable metrics to conclusively demonstrate the efficacy of camera technology in clinical education, continuous performance improvement, and quality enhancement initiatives.

Considering that the primary aim of this study was to compare and recommend a high-quality approach for recording surgical videos, future research will focus on conducting multi-centered studies with larger sample sizes and emphasis on the diversity of surgical specialties and camera brands. It is also essential to assess its application more effectively in a learning experience in surgical education, not only in head and neck surgery but also in other surgical areas. Future studies will improve the evaluation of skill levels through practical techniques and written exams, study learning curves in relation to surgical timing, analyze cost-effectiveness, and gather evaluations from the trainer’s perspective.

## Conclusion

The field of head and neck surgery has consistently welcomed innovation, embracing the introduction of new techniques into surgical practice. There is a substantial demand and room for development in the domain of open surgical recordings. Surgical video recording serves the purpose of technical communication and accomplishes the objective of medical education through real-time connectivity, addressing the current global shortage of specialized surgeons. The two systems examined in this study, the GoPro and the digital camera, each have distinct features and advantages. The GoPro, an affordable and physician-independent solution, offers a stable and continuous view of the surgical area, though it lacks a medical-specific design and a zoom function. On the other hand, despite requiring periodic repositioning and potentially distracting the surgical team, the digital camera delivers superior visibility of anatomical details and higher image quality.

### Supplementary Information

Supplementary Material 1.

Supplementary Material 2.

## Data Availability

The data supporting the findings of this study are available within the article and its supplementary materials.
