# Supplementary material for: Comparative analysis of GoPro and digital cameras in head and neck flap harvesting surgery video documentation: an innovative and efficient method for surgical education
Source: BMC Med Educ. 2024 May 14;24:531. doi: 10.1186/s12909-024-05510-2 (PMC11092002; doi:10.1186/s12909-024-05510-2)
Supplement: Supplementary file 1 — Supplementary Material 1. [file 12909_2024_5510_MOESM1_ESM.pdf]

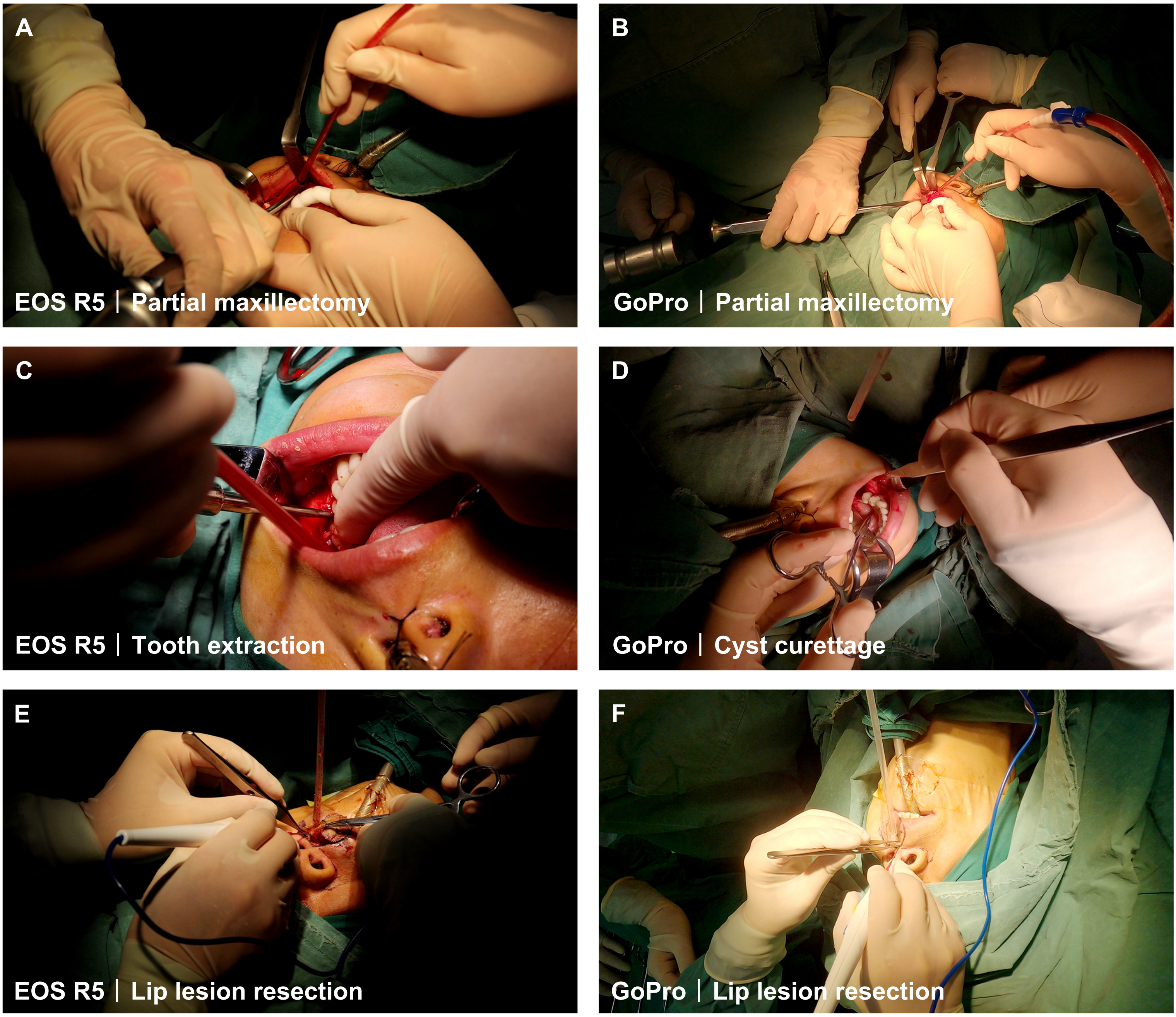


**Figure S1.** Video recordings of other surgical types. A-F show three different surgeries recorded using digital camera (A, C, E) and GoPro (B, D, F) . Digital camera shot the surgical area more accurately while GoPro have a wider field of view.
